# Eruptive Keratoacanthomas Arising Within Eosinophilic Annular Erythema

**DOI:** 10.7759/cureus.108837

**Published:** 2026-05-14

**Authors:** Sahla Esam, Theodore Katz, Nkanyezi Ferguson, Susan M Zurowski

**Affiliations:** 1 Dermatology, University of Missouri School of Medicine, Columbia, USA

**Keywords:** basal cell carcinoma, eosinophilic annular erythema, eosinophilic dermatosis, keratoacanthomas, squamous cell carcinoma in-situ

## Abstract

Eosinophilic annular erythema (EAE) is a rare skin disorder that presents abruptly with erythematous annular plaques. Typically, these plaques spread centrifugally with central clearing and recur in a relapsing-remitting pattern. Histologic findings consist of dermal perivascular to dense eosinophilic infiltrates, with preservation of the epidermis and subcutis. In this report, we describe a unique case in an 85-year-old woman characterized by a chronic course of EAE that was initially localized, along with the development of multiple keratoacanthomas (KAs) arising within the affected skin. The onset of EAE eruption followed biopsy and treatment of a basal cell carcinoma, potentially serving as a triggering event. Histologic analysis was consistent with EAE, characterized by dense eosinophilic infiltrates and lacking flame figures. Multiple KAs arose within these lesions. This case highlights a potential clinical association between EAE and KA development, in which the chronic inflammatory microenvironment from EAE increased the risk of KA development, given that persistent inflammation is a known contributor to tumorigenesis. Management included topical corticosteroids for symptomatic erythema and pain, alongside methotrexate therapy to promote KA regression. Further investigation is warranted to better characterize this relationship and its underlying mechanisms.

## Introduction

Eosinophilic annular erythema (EAE) is uniquely characterized by annular erythematous plaques that develop acutely, with a relapsing-remitting course, and expand centrifugally with central clearing. This phenomenon was first described in 1981 within the pediatric population and has since been characterized in adults. The lesion can range from asymptomatic to pruritic and can last 4-12 months, predominantly affecting the trunk and proximal extremities [1]. Histopathology reveals perivascular to dense eosinophilic infiltrates in the dermis with an unremarkable epidermis and subcutis [1,2]. There is ongoing research into the pathogenesis of EAE. Hypotheses include interleukin (IL)-5-mediated involvement and subsequent recruitment of eosinophils in response to an epidermal stimulus [3]. Treatment options for EAE reported in the literature include topical and systemic steroids, hydroxychloroquine, dapsone, doxycycline, or dupilumab [1-4].

## Case presentation

**Figure 1:**
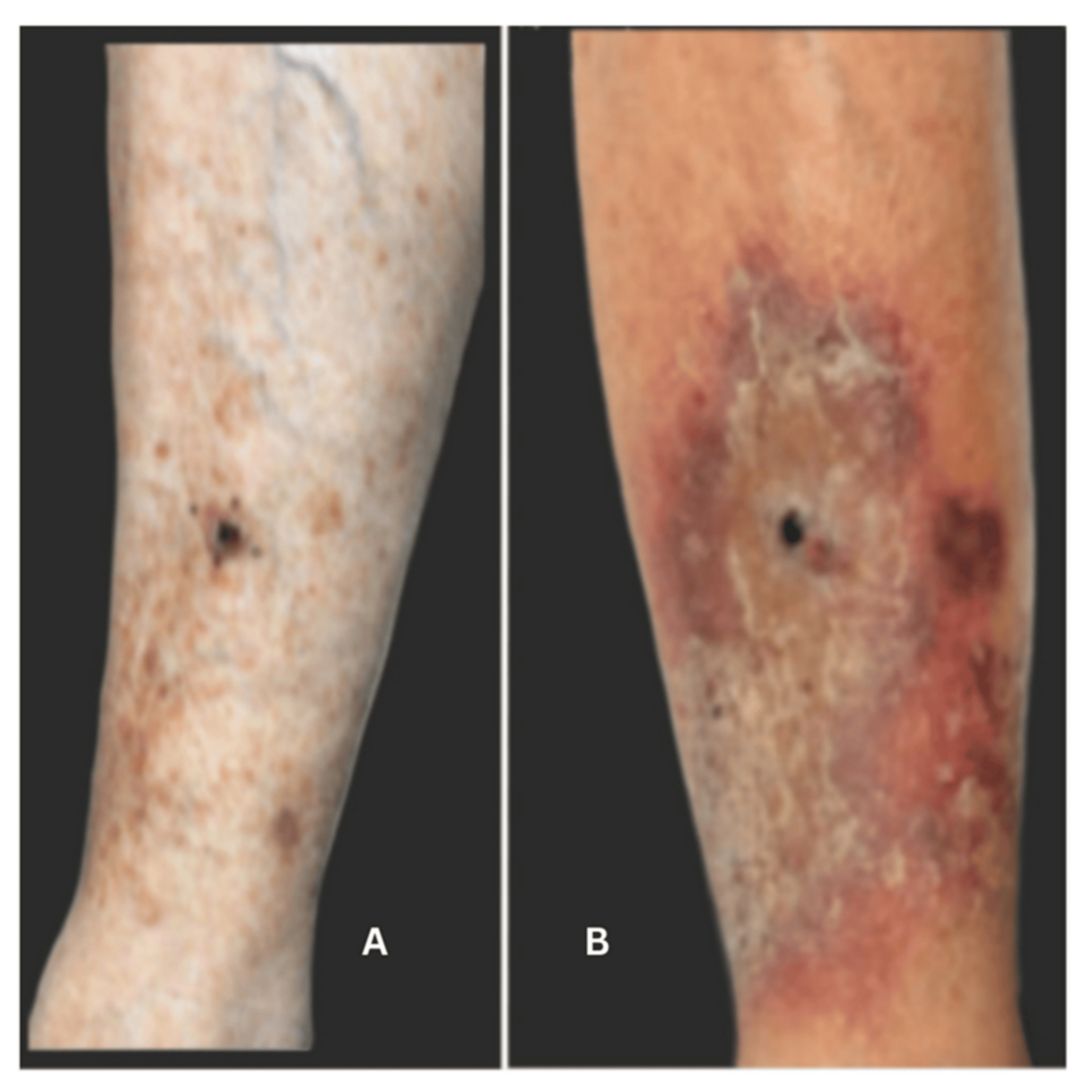
Rash progression (A) Left leg before the onset of the rash, with the BCC marked by four black dots. (B) Rash developing on the left leg shortly after biopsy and destruction of the BCC. BCC: Basal cell carcinoma.

An 85-year-old female with a dermatologic history of keratinocyte skin cancer, including several basal cell carcinomas (BCCs) and one squamous cell carcinoma in situ (SCC), presented to the clinic for a painful, unilateral, worsening rash on the left shin. The rash was centered over a wound from a recent BCC (Figure 1, Panel A) that was destroyed with curettage and cryosurgery two months earlier. Physical examination revealed a plaque with erythematous to purpuric annular borders and a central clearing, along with yellow scale and a hemorrhagic crusted ulcer centered around the recent BCC destruction site (Figure 1, Panel B). Initial treatment with topical mupirocin 2% cream to cover possible infection failed to provide any benefit. Topical triamcinolone 0.1% cream and compression therapy were subsequently recommended, with initial improvement; however, the rash continued to progress with increased erythema at the leading edge.

**Figure 2:**
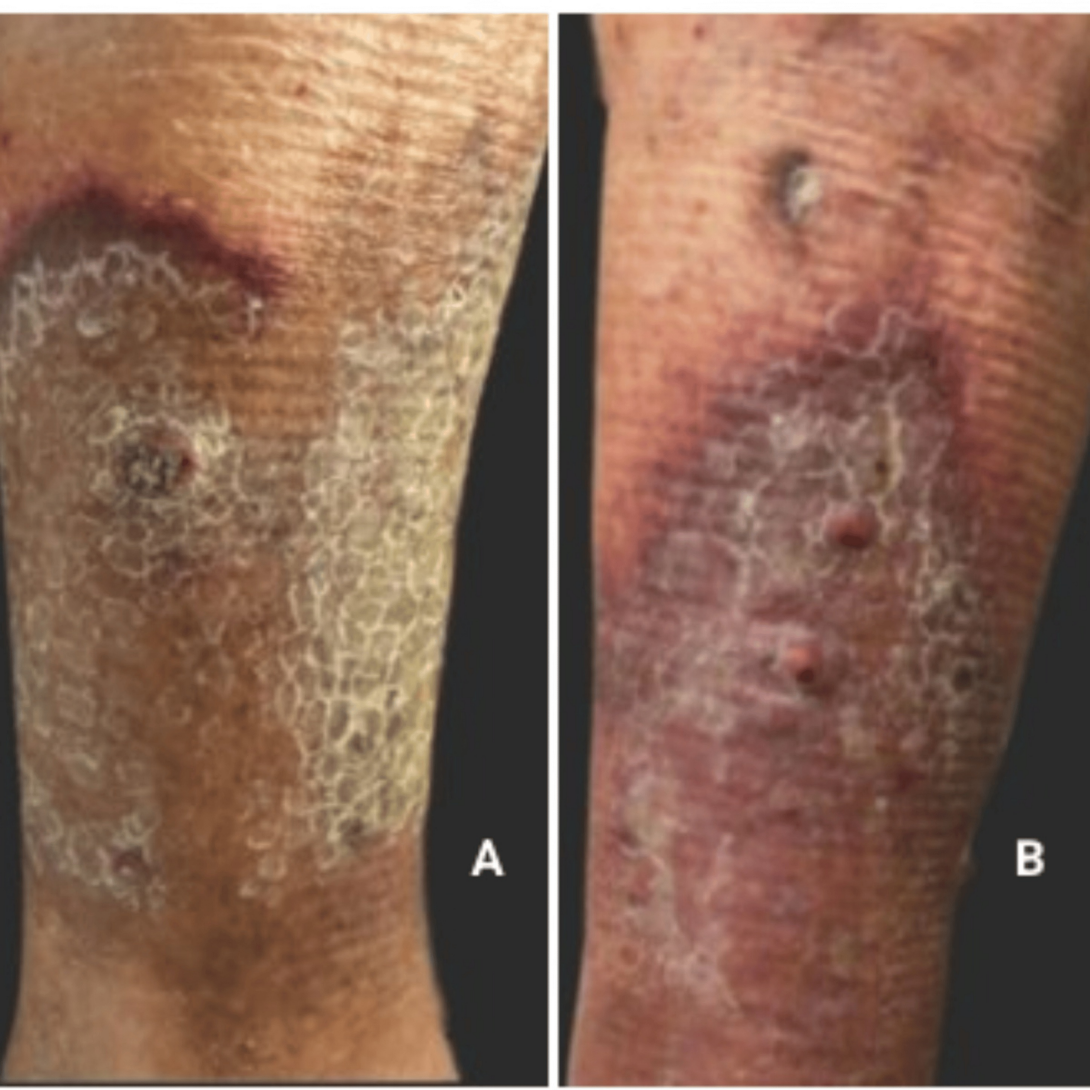
Multiple keratoacanthoma eruptions within the rash (A) A larger keratoacanthoma on the left anterior leg on a background of rash with plate-like scale. (B) A few smaller presumed keratoacanthomas on the anterior right leg within the rash.

After three to four months of an active rash, a keratoacanthoma (KA) was noted within the rash with clinical resolution after a shave biopsy. Several months later, the erythema recurred on the left leg, with a new rash on the right leg as well. Both legs were treated with topical triamcinolone 0.1% cream. Over several months, seven suspected KAs developed within the rash areas, five on the right leg and two on the left (Figure 2). The KAs were treated with one to three injections of intralesional methotrexate, and all lesions clinically resolved.

**Figure 3:**
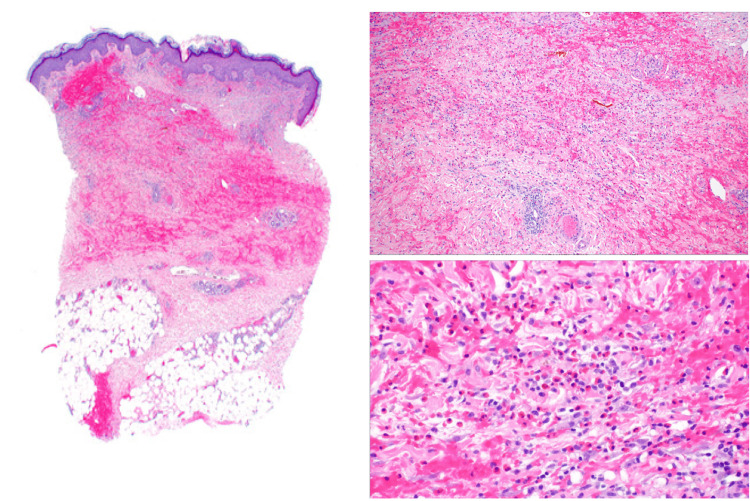
Histopathology (2X, 10X, and 40X magnification) Punch biopsy from the elevated annular erythematous rim shows unremarkable epidermis, hemorrhage, and eosinophils in the deep and superficial dermis, with unremarkable subcutaneous tissue. The architectural overview is shown at 2X magnification, the perivascular/interstitial distribution at 10X, and the high-power confirmation of the dense eosinophilic infiltrate (and lack of flame figures) at 40X.

The erythematous rim of the rash was biopsied, and histopathology showed a dense infiltrate of eosinophils without flame figures and significant associated hemorrhage. Laboratory workup included a complete blood count that revealed no peripheral eosinophilia. Thyroid-stimulating hormone (TSH), complete metabolic panel (CMP), and urinalysis (UA) were all unremarkable. The differential diagnoses included granuloma annulare, stasis dermatitis, pigmented purpuric eruptions, erythema annulare centrifugum, Wells syndrome, and EAE. Based on clinical and histopathological findings, the patient was diagnosed with EAE due to the characteristic dense, superficial, and deep infiltration of the perivascular and/or interstitial tissues with eosinophils (Figure 3).

**Figure 4:**
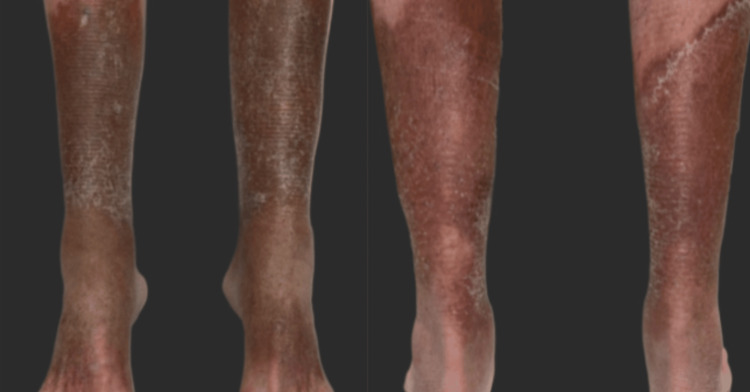
Resolution of lower leg erythema following treatment with halobetasol and compression therapy Anterior and posterior views of the legs following diligent topical treatment with halobetasol cream and compression therapy. Most of the erythema has resolved, with a thin plaque remaining on the right posterior leg. Striking, well-demarcated residual hyperpigmentation is evident.

We recommended subcutaneous dupilumab, as prior reports showed improvement with this well-tolerated treatment, and discussed additional options including oral and topical steroids, compression, and oral hydroxychloroquine. The patient elected to continue treatment with topical steroids (halobetasol 0.05% cream applied BID) and compression garments. This treatment improved the erythema and pain. The residual hyperpigmentation remained unchanged (Figure 4).

## Discussion

EAE exhibits a relapsing and remitting course, is often associated with discomfort, and is typically considered a benign condition. In EAE, an unknown antigen is hypothesized to cause hypersensitivity. Several chronic diseases have been associated with EAE, including diabetes, hepatitis, hematological disorders, and autoimmune conditions such as lupus and rheumatoid arthritis [4]. EAE has also been associated with internal malignancies, with specific malignancies reported in three cases, including thymoma with complete resolution following thymectomy, metastatic prostate adenocarcinoma, and renal carcinoma [5]. We propose that postprocedural trauma served as the inciting factor for EAE in our case, as the condition developed at the BCC site. As eosinophils appear to play a central role in the histopathology of EAE, systemic steroids are the gold standard treatment because of their ability to inhibit eosinophil migration and the release of proinflammatory cytokines [6]. Dacy et al. reported that dupilumab, an IL-4 inhibitor, induced remission in two cases of EAE [6]. Dramatic improvement was observed after two injections, with resolution of all lesions and associated pruritus [7]. Given the eosinophilic density and potential IL-5 involvement, mepolizumab (an IL-5 inhibitor) may also be considered as an additional treatment option [7].

Our patient's clinical course was complicated by multiple KAs. A KA is a skin tumor characterized by a central keratinous plug. KAs typically demonstrate rapid initial growth followed by stabilization and, in some cases, spontaneous regression. Several possible etiologies have been described, including ultraviolet radiation exposure, carcinogen exposure, immunosuppression, and local trauma. Additionally, chronic inflammation has been recognized as a potential contributor to tumorigenesis. In this case, we suspect that the chronic eosinophilic inflammatory milieu associated with EAE may have contributed to the development of these KAs, although a definitive causal relationship cannot be established. Given the patient’s overall clinical presentation and lack of concerning systemic findings, an extensive malignancy evaluation was not pursued. The patient was informed about both localized and systemic treatment options, including intralesional methotrexate and dupilumab, and ultimately elected to proceed with localized therapy using intralesional methotrexate. A case series demonstrated that intralesional methotrexate was associated with regression in 83%-100% of patients with KAs [8]. In our patient, all observed KAs fully regressed following intralesional methotrexate therapy.

## Conclusions

This report describes a unique presentation of chronic EAE associated with the development of multiple eruptive KAs arising within the inflammatory eruption. The rash began following biopsy and destruction of a BCC, which may have acted as a triggering event for the subsequent inflammatory process. Histopathology supported the diagnosis of EAE, demonstrating a dense eosinophilic infiltrate without flame figures. Clinically, prominent hyperpigmentation was observed, likely reflecting post-inflammatory changes, hemosiderin deposition, and a possible component of superimposed stasis. Multiple KAs subsequently developed within the involved inflammatory fields; one lesion involuted following biopsy alone, while seven others resolved after treatment with intralesional methotrexate. The clinical chronology and histopathologic findings raise the possibility of a rare inflammation-associated tumorigenic process occurring within a chronic eosinophilic inflammatory microenvironment. However, given the limitations of a single case report, this association should be interpreted cautiously and not as evidence of a definitive causal or pathogenic relationship. Further studies are needed to better characterize any potential link between chronic eosinophilic inflammation and KA development. Although not used in this patient’s treatment course, dupilumab may represent a potential steroid-sparing therapeutic consideration in future cases of refractory EAE given its emerging role in eosinophilic dermatoses.
